# Mapping the cultural characteristics of subcontinent students and the challenges to their academic success

**DOI:** 10.1371/journal.pone.0272159

**Published:** 2022-08-03

**Authors:** Ritesh Chugh, Stephanie Macht, Monika Kansal, Robert Grose, Mahsood Shah, Anthony Weber

**Affiliations:** 1 School of Engineering and Technology, Central Queensland University, Rockhampton, Queensland, Australia; 2 Centre for Research in Equity and Advancement of Teaching and Education, Central Queensland University, Rockhampton, Queensland, Australia; 3 School of Business and Law, Central Queensland University, Rockhampton, Queensland, Australia; 4 Swinburne University of Technology, Sydney, New South Wales, Australia; Fiji National University, FIJI

## Abstract

This study explores the cultural characteristics of subcontinent students and maps the characteristics to the challenges to their academic success. Interviews of fifty staff from an Australian university indicated that both teaching and professional staff held similar views on the characteristics of subcontinent students. Significant characteristics included respect for teachers, the need for continual guidance, a tendency to group, and a propensity to negotiate. The identified challenges to the academic success of subcontinent students were a lack of engagement with staff, inadequate critical thinking, poor communication skills, academic integrity issues and unrealistic expectations. Armed with a better understanding of the subcontinent student cohort, this study encourages teaching and professional staff to find ways to develop a more inclusive educational environment that builds students up for success.

## Introduction

The number of students worldwide travelling abroad to further their education has increased exponentially over the past few decades [1, 2]. In terms of the total number of foreign and international students in tertiary institutions, Australia sits fifth behind the United States, the United Kingdom, Germany and France [3]. In 2017, over 624,000 international students were studying in Australia [4].

There is an abundance of literature highlighting the challenges faced by international students. For example, Wang and Shan [5] noted that international students have difficulty adapting to conventions associated with academic integrity, while Kingston and Forland [6] noted that many international students are stressed and confused when confronted with alternative teaching and learning styles. Another study found that higher levels of acculturative stress were reported by international students from Asia [7]. However, Gunawardena and Wilson [8] warned against treating international students from different countries under the same lens. By focusing on only one group of international students, which has hitherto been neglected in the literature, the current paper addresses this warning, and separately considers only students from the subcontinent and their learning and engagement styles, as shaped by their specific cultural contexts. South Asian countries include Bangladesh, Bhutan, India, the Maldives, Nepal, Pakistan, and Sri Lanka, and collectively they are also referred to as the subcontinent. Between 2012 to 2017, Australia witnessed a significant increase of 37,195 students from the subcontinent [9]. Hence, to improve academic success and retention of this expanding group, it is necessary to understand their cultural characteristics and identify the challenges they face in achieving academic success.

Recent media reports suggest a rise in mental health and stress issues among international onshore students because of financial pressures, navigating a new culture, and adjusting to a new academic system [10]. Under this type of pressure, international students usually adopt the learning approaches used in their native culture, which may not best serve their interests [11]. The continuing growth of students from subcontinent backgrounds necessitates that higher education institutions consider providing ongoing professional development for staff regarding cross-cultural issues, and develop suitable strategies to engage with international students.

Riley and Ungerleider [12] suggested that teachers’ misconceptions about students can restrict students’ effective academic development. Given that past and present students represent a key source of marketing and recruitment of prospective students [13], a lack of appreciation of cross-cultural issues could cause significant reputational and economic risk to the university sector in Australia and elsewhere. Given this background, it is critical that academics within the university sector fully appreciate the cross-cultural issues affecting international students, and develop a region-specific professional development programme [14] to help academic staff better understand the unique needs of subcontinent students [15]. Miles and Leinster [16] called for further research to help teaching staff better appreciate the challenges faced by international students, rather than relying on preconceived (and often incorrect) ideas about their learning habits. Having a better awareness of the learning-related cross-cultural issues of international students (from subcontinent countries) would allow for improved pedagogy and improved student retention, which should ultimately result in improved student success rates [17]. In considering learning and student success theories, it is vital to consider cultural perspectives and their impact on learning and teaching. Vygotsky’s sociocultural theory outlines the significance of culture in learning and that learning and culture are interdependent [18]. Sociocultural theory provides a theoretical framework for understanding such learning environments.

Previous studies have looked at international students collectively but not at the subcontinent student cohort separately. To the best of the researchers’ knowledge, prior studies have not explored the challenges related to the academic success of subcontinent students posed by cross-cultural issues. This study fills the research vacuum by not only creating a better understanding of the characteristics of students from the subcontinent but also mapping how these characteristics translate into challenges to their academic success. Moreover, previously, the collective views of teaching and professional staff have not been explored. Focus groups were conducted to understand the challenges to the academic success of subcontinent students due to cross-cultural issues. The research outcomes will enable university academic and professional staff to better engage and support subcontinent students, ultimately leading to academic success. The two research questions (RQ) that this study answers are:

RQ 1: What are the cultural characteristics of subcontinent students?

RQ 2: How do these cultural characteristics present challenges to the academic success of subcontinent students?

## Literature review

Literature about the challenges faced by international students abounds [19–21]; however, much of this literature examines the challenges from a student perspective. Literature about the challenges faced by teaching and professional staff when dealing with international students is much sparser [20]. To create a culture of academic success in a learning setting, the constructs of learning, achievement, resistance and success play a significant role [22]. Sociocultural approaches to learning and development are based on the belief that human activities, such as learning, take place in diverse cultural contexts and are influenced by language and thought [23]. Hence, to make students the centre of the learning process, it becomes important to explore the cultural characteristics of students to drive their success in the educational journey.

From an academic perspective, there is often an expectation that international students should deal with the learning environment in the same manner as host country students. However, international students have difficulty adapting to a student-centred approach to learning; thus, many adopt a ‘head in the sand’ approach until it is too late for corrective action [19]. Many university academics lack a sufficient understanding of why students struggle to adapt to a new country and its learning culture [24]. Consequently, it becomes crucial to understand the challenges to academic success and how cultural traits can also influence success. It is not just the academic learning environment that can cause a misalignment or lack of understanding of the plight of international students. Many academic staff held the view that students would often actively seek to segregate themselves into groups with others of their cultural background [25]. Similarly, support staff expressed concerns about their inability to facilitate the integration of international and host students [20]. A lack of understanding of the personal challenges confronting students often causes teachers to develop negative perceptions or stereotype these students.

Given that there is a significant amount of literature discussing the difficulties faced by international students [19–21], it is unsurprising that teachers tend to focus on the negative aspects of this student cohort. However, this unhealthy approach hides the positive experiences of international students studying abroad [20, 26]. Unfortunately, the existence of a significant volume of negative literature may have biased the views of teaching staff, and may be responsible for the development of a negative perception of international students in terms of their ability to adapt to the learning environment of the host country [6, 19, 27]. However, to assist students be more successful, it is important to be aware of their cultural characteristics and behaviour patterns [28].

Trice [26] observed that the literature between 1984 and 2003 demonstrated a gradual deterioration of staff attitudes towards international students in the United States and the United Kingdom. However, it should be noted that this was not a deterioration in general, as the benefits of having cultural diversity in the classroom were highly praised by staff, although they were conscious of the challenges associated with teaching and supporting international students because of language, culture and other reasons. Somewhat comforting was the acknowledgement that staff need to be prepared to support international students and increase their cross-cultural learning [29]. Cultural identity plays a vital role in academic achievement and success; a need to increase awareness of cultural values exists, and future research can examine identity in other cultural contexts [30].

Although international students are required to provide evidence of their language proficiency in terms of their reading, writing, speaking and understanding of English, issues and challenges remain [31, 32]. Unfortunately, many international students do not possess a sufficient grasp of English; thus, their learning capacity, ability to achieve above-average grades, ability to participate in meaningful group discussions, and ability to interact with teachers and local students is severely compromised [33–35]. O’Reilly et al. [20] also discussed the many frustrations experienced by professional staff related to language difficulties and cultural barriers. A number of ‘dichotomies’ are mentioned in the literature, which international students must navigate [19], including:

- ‘being taught’ (home country) versus ‘independent study and critical thinking’ (host country)
- ‘needing to be shown’ (home country) versus ‘simply being told’ (host country)
- teachers being perceived as experts and unapproachable by students (home country) versus teachers as facilitators who wish to appear approachable and friendly (host country).

O’Reilly et al. [20] highlighted the perception by some professional staff that international students are ‘demanding’ because they can ask many questions in rapid succession. The researchers suggested that these frequent communications may be a result of students not being (fully) aware of the interaction styles typically used in their host country. Kingston and Forland [6] mentioned that many students not only struggle with language and cultural adaptation issues, but also struggle with a loss of confidence as a result of a combination of various other struggles. Given these struggles, it is perplexing that international students choose not to avail themselves of the many academic support facilities available to them, even though it would be useful for their skills development.

Moreover, the sociocultural theory suggests that learning is a social process with the learning environment playing a critical role in learner development [36]. Hence, an identification of the different elements of learning, such as social characteristics, communication styles, and cognition, is vital, especially as these elements develop from social and cultural connections as outlined in Vygotsky’s sociocultural theory [37]. As a constructivist theory of learning, the sociocultural theory is particularly well suited to examining the challenges to the academic success of students from a learning perspective and the characteristics of subcontinent students from a cultural context. However, there remains a lack of sufficient evidence in demonstrating the cultural characteristics of subcontinent students that could potentially mature into academic challenges. This study fills the gap.

## Research method

This study employed focus groups as a qualitative research method for data collection. Focus groups help in developing a common understanding of the shared problems and solutions thereof in complex psychosocial phenomena [38]. Thus, they are best suited for comprehending the complexity of staff’s perceptions about the cultural traits of the subcontinent students and challenges to student academic success. Focus groups use and encourage communication among several participants simultaneously to generate data to examine, clarify, and explore their knowledge and experiences [39].

Eight focus groups were conducted; four with teaching staff (also referred to as academic staff) and four with professional staff from a regional Australian university. The total number of participants (n = 50) comprised 29 academic and 21 professional staff, averaging 6 to 7 participants per group. The academic cohort included a mix of both full-time and casual teaching staff who taught large numbers of subcontinent students. In contrast, only full-time professional staff were interviewed, as the university did not employ many casual professional staff. Professional staff for the focus groups were recruited from a diverse range of areas, such as student services, counselling and advocacy, student governance, academic support and international student support; thus, they included a good spread of participants who were at the frontline of supporting subcontinent students. The recruitment of professional staff in this manner ensured that participant selection was homogeneous and based on variables that were designed to yield important information [40]. The focus groups were run face-to-face by experienced members of the research team at three campuses of the university in three states. The facilitators ensured that all participants had sufficient opportunity to share their views freely by creating a relaxed and friendly environment.

Four focus groups are usually considered adequate for research completion [40]. However, because this study involved two different cohorts (teaching and professional staff), eight focus groups provided thoroughness and enabled the capture of richer perspectives. Furthermore, Guest et al. [41] asserted that three focus groups were enough to discern ninety per cent of themes and attain saturation, if the respondents were not quite heterogeneous. After conducting four focus groups in each category, we could not find any new insights from the data due to data saturation; thus, the team decided to conclude data collection. Participants who had at least some exposure to subcontinent students were recruited via an email invitation, accompanied with an information sheet and a consent form. The study did not employ any exclusion criteria nor collected any demographic details of the participants. However, the staff-diversity policy of the host university helped ensure that the participants had demographic heterogeneity as well as sufficient homogeneity in educational and occupational backgrounds. Two separate sets of questions (for teaching and professional staff each) were developed de-novo and pilot-tested with peers. The focus group questions related to unique classroom behaviours, if any, demonstrated by students from the subcontinent and the academic challenges faced by them due to their culture-based characteristics. The participants were prompted to provide examples wherever appropriate. Each focus group lasted for approximately one hour.

This research was approved by the host university’s ethics committee (Approval Number: 0000021254), and all ethics procedures with regards to informed consent, withdrawal, and assurance of anonymity at all stages of the research process were followed. Prior to the focus group, potential participants were emailed an information sheet with details of the study and a consent form. Written informed consent from all participants was obtained before they participated. Staff who agreed to participate emailed back a signed consent form, which signalled their indication to participate. With the consent of the participants, the focus group sessions were recorded and de-identified with a number assigned to each participant and focus group; with direct quotes referenced as FG1 (Focus Group 1)_AS (Academic Staff) or PS (Professional Staff)_P1 (Participant 1).

**Fig 1:**
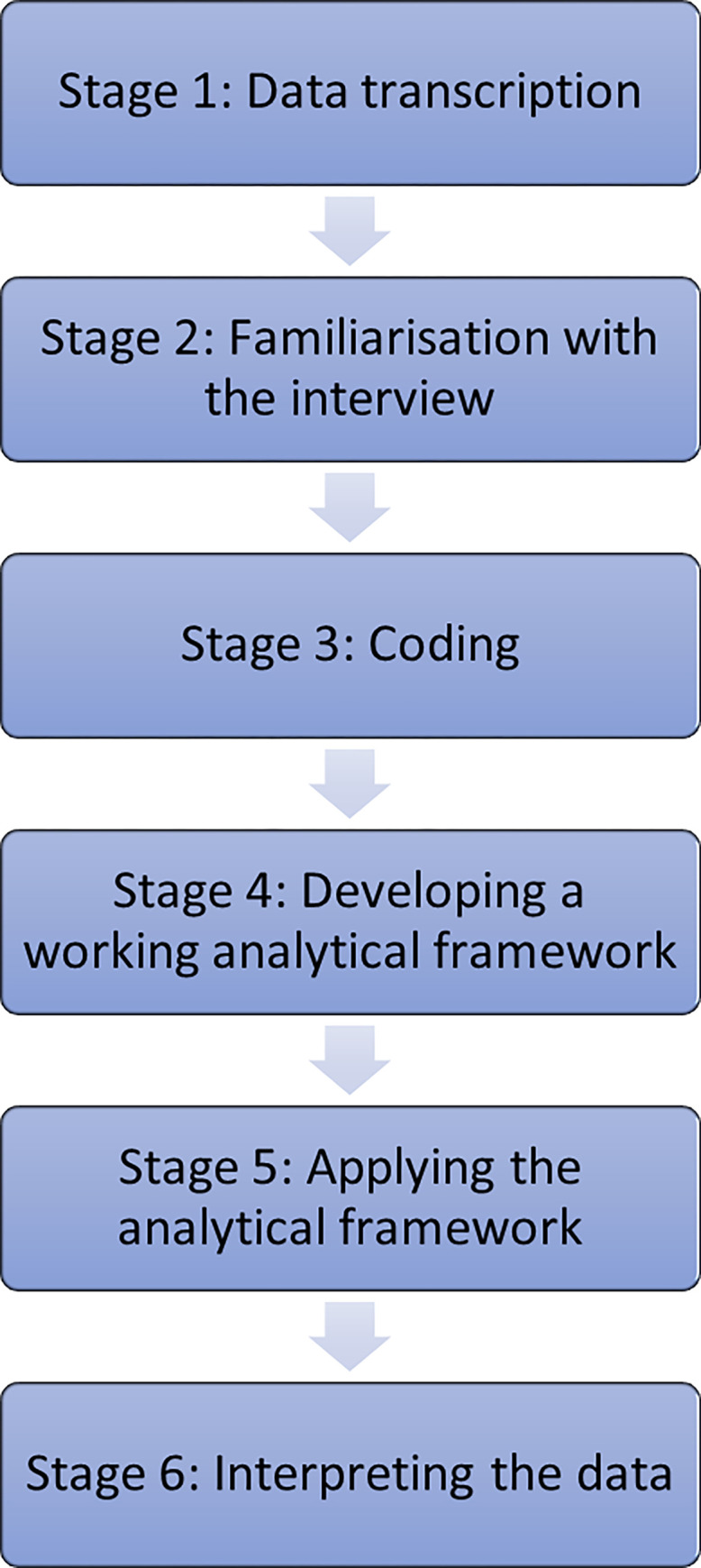
Framework method for data analysis.

The qualitative data analysis involved summarization, classification, and interpretation to obtain elaborate responses to the questions of interest [42]. This study used ‘the framework method’ as its systematic procedure is suitable for multidisciplinary teams and/or with large datasets, and is easy to follow [43]. Fig 1 depicts a succinct adaptation of the Gale et al. [43] framework method. The data were transcribed verbatim by a professional transcriptionist. The data collection questions provided the initial code structure, which evolved with an inductive coding approach [44]. Experienced researchers in the team conducted the focus groups to ensure data familiarisation and reliability. Two research team members initially read and independently coded two transcripts from each set of participants. The initially identified coding schemes of the two researchers were compared for consistency, ensuring further data reliability, with no text reduction at this stage. Once the coding structure and analytical framework were finalised, the text in each manuscript was manually organised and classified under coding themes using NVivo qualitative data analysis software. Emerging themes were categorised using constant comparative analysis—a repetitive process of grouping similar ideas and seeking new themes in the qualitative data [45]. Finally, interpretation of data was undertaken, which involved summarising the data by category from each transcript to attain a balance between data reduction and retaining the original meaning of the participants’ words [43].

## Findings and discussion

The focus groups sought responses to better understand the challenges to the academic success of subcontinent students due to cross-cultural issues, as identified by both academic and professional staff. Therefore, it is necessary first to identify the perceptions of staff about the characteristics of subcontinent students before mapping the characteristics to the challenges to their academic success (summarized in Table 1).

**Table 1:** Staff perceptions: Mapping student cultural characteristics and challenges to academic success.

*Subcontinent student characteristics*	*Terms used*	*Challenges to academic success*
Respect for teachers	Guru, master, weighty, honourable, transformative-figure	Lack of engagement with staff
Guidance dependency	Spoon-fed, laid out on a plate	Poor critical thinking skills
Tendency to group	Security in the cultural group, attachment, community, belongingness, cultural ghetto	Inadequate communication skills and academic integrity
Master negotiators	Ingrained life-skill, win-lose proposition	Unrealistic expectations

### Staff perceptions of subcontinent student characteristics

Both teaching and professional staff held similar views on the characteristics of subcontinent students. Four significant characteristics—excessive respect for teachers, guidance dependency, a tendency to group and a strong propensity to negotiate are discussed in the following paragraphs.

#### Respect for teachers

Respect for teachers is a cultural trait of many subcontinent students. The focus group participants collectively shared the notion that subcontinent students regarded their teachers as transformative figures, and subsequently, had a very strong emotional attachment to their teachers. The subcontinent students regarded themselves as having a lower level of status than their teachers and were respectful based on their position in the hierarchy:

*We have differences which is culturally situated around things like our distance and respect for elders*, *and education traditions as well*, *and I think generally Asian traditions are generally that you learn from the master*. *You learn from a sage*. *You learn from a guru*. *And you’re the student*, *you’re at a much lower level*, *whereas perhaps the Anglo-American tradition is more a questioning*, *challenging and critical thinking tradition*. (FG1_AS_P2)

Many focus group participants concurred when one professional staff member expressed surprise over the level of respect afforded to lecturers: *‘They keep calling them professors*, *for a start*. *“My professor*, *my professor*!*”‘* (FG5_PS_P1). A participant suggested that students were shy and did not want to say anything that contradicted their teacher, while another professional staff member commented that *‘they would not ask questions*, *and they will be extremely respectful of their lecturers*.*’* (FG6_PS_P1). One problem associated with this deference to authority is the tendency for subcontinent students not to engage with teachers in an enquiring manner. The findings of the study are supported by Marambe et al. [46] as they also identified respect for teachers as a prominent behaviour of Sri Lankan students. They argued that such respect is embedded in the definition of the term ‘Guru’ contained in the word ‘Guruvaraya’, or teacher or a person who is ‘weighty’ or ‘honourable’. These culturally derived attitudes are in stark contrast to the typical requirements of the Australian higher education system, where students are expected to function without much guidance and consider the teacher as a facilitator rather than an authority figure [47].

#### Guidance dependency

The flow-on effect of excessive respect for teachers may translate to subcontinent students often relying on exclusive guidance from the staff. Most focus group participants perceived that many subcontinent students exhibit ‘teacher guidance dependency’ behaviour. This is because their prior learning experience emphasises teachers as the principal vehicle for learning rather than facilitators of learning in the independent learning environment in Western cultures. Most of the focus group members affirmed the statement by one professional staff that subcontinent students were accustomed to being ‘spoon-fed’. The focus group excerpt is as follows:

*Students come with the culture of being spoon-fed*. *They want everything laid out to them on a plate and they don’t want to work towards it*. *They want everything done for them*. (FG6_PS_P5)

This lack of self-initiative or an inability to take control of their learning often has significant implications on students’ learning experience and academic success when preparing for a final examination, which requires self-initiative and independent thinking without specific guidance on exam content. One participant commented:

*So here [in Australia]*, *…the students have the equation*, *you are not testing on memory; rather*, *you are testing … how would you apply this equation in a situation*? *That’s true learning*. *So that’s absent in many of the subcontinental curricula and methodology of teaching*. (FG2_AS_P4).

#### Tendency to group

Students will sometimes form groups with other students from a familiar country or context for comfort or security reasons. The participants provided clear feedback on the phenomenon of subcontinent students seeking associations only with their own kind. One professional staff participant suggested that a possible explanation for this inclination related to the students’ preference to be with people who could help them when and where needed:

*In my interactions*, *I’ve found they’re quite attached to their community or if they’ve got family*, *so they want to feel like belonged when they go to a place*, *they will have a community to fit into that will help them*. (FG8_PS_P1)

Perhaps the strongest reference to this phenomenon was the use of the term ‘cultural ghetto’, as one participant described it: *‘But*, *it’s the cultural ghetto*. *They do not have anyone else to go to—they’re not integrated with local students’* (FG3_AS_P2). This tendency to stick together may mature into a serious academic concern when students collude in the production of group assessments [48]. Further, these students seek advice from their cultural peers on course and assessment issues, rather than gathering relevant information through independent research. This second-hand information has the potential to mislead students to make incorrect decisions about critical assessment matters. Earlier literature by Crozier et al. [49] and Trice [25] also notes that students from Asian cultural backgrounds sought the company of their own cultures as a means of sharing their personal experiences and possibly as a source of security.

#### Master negotiators

Many cultures consider the ability to negotiate an important and ingrained life skill. The focus group participants asserted that subcontinent students in general, and Indians specifically, tend to attain specific outcomes from their negotiations rather than achieving a broad agreement. They described this negotiation aggressiveness as a benign cultural characteristic of subcontinent people that needs to be understood and accepted:

*It’s not an aggressiveness*. *It’s a cultural way of operating which you [students] have to adopt to*, *to be effective in their society … So*, *okay*, *you [university staff] have just got to deal with it*. (FG1_AS_P5)

Many focus group participants have experienced such negotiating tendencies in their role as academics or professional staff in the following three areas. First, in the assessment of grades, particularly if the allocated mark was below the coveted pass benchmark. Second, negotiation was sometimes used as a tactic to impede plagiarism proceedings. Finally, the subcontinent students used negotiating skills when it was time to pay tuition fees. As one professional staff member lamented: *‘Negotiation*. *Negotiation is a thing that*, *let’s say*, *70% of those students that I see in regards with fees*, *“Can we negotiate payment deadline*?*”* (FG6_PS_P2).

At a cultural level, some of O’Reilly et al.’s [20] respondents highlighted examples of the bargaining mentality of international students—that is, many international students tend not to accept a ‘no’ response from teachers, even in situations where the reasoning for such responses is obvious and clearly explained.

### Challenges to the academic success of subcontinent students

The identified characteristics of subcontinent students present challenges for developing appropriate study skills and attaining academic success. The participants stressed that common cultural characteristics of subcontinent students impede the progress of relevant learning competencies such as communication, independent thinking skills and active engagement with staff, culminating into suboptimal academic success.

#### Lack of engagement with staff

‘Unwilling’, ‘shy’ and ‘scared’ were terms that participants used to describe the resistance of subcontinent students to engaging and participating in their learning journey. Excessive respect for teaching staff and seeing them as ‘Guru’ may lead subcontinent students to impetuously accept the knowledge as delivered by the teachers rather than counter-questioning and clarifying their doubts by actively engaging with them. Many participants recorded their consensus when one academic participant stated: *‘They feel shy to ask questions*, *and they never contradict their lecturers*. *That’s one of the biggest problems’* (FG2_AS_P4). This apparent shyness may relate to poor communication skills and passive learning tendencies. It is encouraging to find similar results by Safipour et al. [34], who inferred that language inadequacy combined with learning cultures that do not promote active participation can bring about suboptimal academic outcomes for international students.

#### Insufficient critical thinking

Undue dependency upon guidance provided by teachers may result in inadequate critical thinking skills or independent thinking. Universities in Australia (and elsewhere) expect that students should be able to think critically. Critical thinking is often referred to as ‘reasonable reflective thinking focussed on deciding what to believe or do’ [50]. In this study, when referring to critical thinking, many teaching staff participants used the term ‘independent thinking’, rather than ‘critical thinking’. However, both teaching and professional staff perceived that subcontinent students generally failed to show adequate critical thinking behaviour or a confident or well-thought-out conclusive decision. Lack of this crucial capability diminishes their likelihood of academic success. For example, one participant mentioned that many subcontinent students had never written an essay, while another commented that students rarely read all instructions. One academic staff member stated:

*But they do expect a lot of directions in many cases*. *They don’t understand the independent study units especially*. *Because they don’t think that they have to actually read all the instructions and come up with their own process of learning*. (FG1_AS_P1)

A professional staff participant also confirmed this lack of independent thinking: *‘So*, *they’re waiting for someone to tell them what to do*, *rather than work their own way’* (FG8_PS_P2). Our results find support from a previous study by Loh and Teo [47] that students from similar cultural backgrounds demonstrate guidance dependency and face difficulties when exposed to Western learning styles. Furthermore, such students often find the facilitating role of educators to misalign with their expectations of a structured, detailed delivery of course content.

#### Deficient communication skills and academic integrity issues

The participants stated that the ‘tendency to group together’, a commonly observed cultural trait of subcontinent students, may presage two challenges to their academic success; first poor communication skills, and second, academic integrity issues. Their cultural preference to clique with students from their own cultural background makes them lose the chance to advance their verbal comprehension and writing skills through interactions or group work with their Australian classmates. The participants almost unanimously stated that insufficient language skills are a pressing academic issue with subcontinent students. One academic staff participant stated that the accents of many subcontinent students were of concern. *‘Additionally*, *there’s the issue of accents*. *The students might have accents which limits the lecturer’s ability to understand*. *That’s the first issue—language*.*’* (FG1_AS_P8). Regarding written communications skills, the following comment on miscommunication is poignant:

*‘they might read policy*, *but they don’t comprehend what’s the meaning of it*. *For example*, *in English*, *the word ‘may’*, *‘I may come’—it’s translated in their own language to ‘you may or may not’*. (FG2_AS_P6)

Both academic and professional staff presented a collaborative view that subcontinent students’ inclination to group with their cultural peers may present serious academic integrity issues. In terms of academic integrity, the main area of concern in the current study related to plagiarism, including both copying of other people’s work and the use of references without proper attribution. Differences in writing styles and the educational culture of the international students’ home country may be a factor in the rise of plagiarism cases involving international students [51]. Participants perceived that students do not necessarily intend to cheat, but a lack of understanding or awareness of what constitutes plagiarism is the primary reason for committing the offence: *‘It’s [plagiarism] not uncommon*, *but I think*, *in many cases*, *it’s just the lack of understanding of what constitutes plagiarism*, *instead of an intention to commit a crime’* (FG1_AS_P1). Chien [52] argued that the proliferation of plagiarism cases by non-English-speaking students is probably related to their low language proficiency, lack of education about writing essays, and differing approaches to citation and referencing styles. Pairing with students outside of their cultural community may provide them with a better understanding of plagiarism and academic integrity as in the Australian education system the concept of plagiarism is instilled at an early age. One participant emphasised that:

*In those countries [subcontinent]*, *plagiarism is not taught at the very outset of the course*. *Here [in Australia]*, *it’s right from Grade 1*, *Grade 2*, *Grade 3*. *So*, *the students know about what is to be done*, *what is not to be copied*, *and how to cite references*. (FG2_AS_P6)

Professional staff also appeared to express concern about the increasing instances of contract cheating, which involves students paying to complete assessments for them: *‘From my experience*, *this cohort is more prone to cheating or exploring options of cheating’* (FG8_PS_P2). A lack of academic integrity was mentioned as a significant challenge to the academic success of subcontinent students by academic staff. Earlier literature by Al-Shamaa et al. [48] and Bretag et al. [53] supports the findings of this study in proposing that international students were found to be largely unprepared for the requirements of producing original work and avoiding plagiarism as compared to domestic students.

#### Unrealistic student expectations

The negotiating propensities of subcontinent students may translate into their heightened expectations for instant service from professional staff and aggressive bargaining with academic staff for pass marks irrespective of their academic merit. Both academic and professional staff cited a range of unrealistic student expectations they had to contend with when working with subcontinent students. These unrealistic expectations included the habit of not purchasing textbooks and other materials and still expecting to pass, not recognising that fifty is the passing grade and not a basis for negotiation, not being satisfied with the outcome of an administration-related academic integrity process, and often demanding immediate outcomes.

Many academic participants shared how subcontinent students expected them to add marks to their grade, without any further effort from the student. For example:

*When the student got 45 or 44*, *and then six more marks would pass them*, *it looks simple to them*, *but I explain that I’ve called them for an interview and explained to them—look*, *this is the criteria and we are appointed academics to ensure that you have achieved the learning objectives*. (FG2_AS_P6)

## Conclusion

The findings presented in this study provide a unique perspective of the cultural characteristics of subcontinent students potentially maturing into significant academic challenges. The key contribution of this study is that it examined these challenges from the perspective of those who teach and administer the needs of international students, including students from subcontinent countries. The study presented the findings under two themes: views on the characteristics of subcontinent students, and then mapping these characteristics to the challenges to academic success.

We found that the high degree of respect displayed by subcontinent students towards academic staff and excessive guidance dependency leads to a lack of active engagement with staff and low development of critical thinking skills as they fail to ask subjective questions during class discussions. A non-enquiring attitude does not fit the Australian (or other Western) academic environment, which demands the use of questioning, challenging and critical thinking skills. We found that academic staff were exasperated by the tendency of subcontinent students to mix only with students from the so-called ‘cultural ghetto’ and failing to properly experience the cultural niceties of their domestic peers. Such tendencies pave the way for collusion in group assessments and seeking academic sub-standard or second-hand advice and direction from their cultural groups. Staff was disappointed over the subcontinent students’ thought process that it is acceptable to negotiate for better marks and payment deadlines. While some staff understood that this tendency to negotiate as part of the students’ cultural upbringing, others felt agitated over such behaviour. Subcontinent students may rely upon their bargaining skills to attain their desired grades through aggressive (and fruitless) negotiations with teaching staff. Thus, staff perceives that the cultural characteristics of subcontinent students expose them to serious challenges to their academic success.

This study has policy implications for universities and the staff therein. Firstly, universities may endeavour to address the identified challenges to the academic success of subcontinent students by equipping and training their academic and professional staff to better cope with the unique cultural behaviours of their students. Furthermore, based on the socio-cultural theoretical lens, the results of this study may encourage teaching and professional staff to develop more inclusive pedagogical settings, and provide more targeted support strategies for subcontinent students, for instance, conscious designing of interventions that engage subcontinent students with their domestic peers and university staff.

This study is not free from limitations. The first limitation is that the participants were not dealing exclusively with students from the subcontinent but with international students in general, in their roles as academics or professional staff. To overcome this limitation, the focus group facilitators constantly reminded participants that their discussions should focus on subcontinent students. Secondly, the findings from this study should not be generalised to the entire international student body, as the focus was only on students from the subcontinent. Similar studies can be conducted to explore cross-cultural challenges in teaching and supporting international student cohorts from other regions, such as Latin America and Europe. Furthermore, although a sufficiently large number of participants were part of the focus groups, the study was conducted at one Australian regional university; hence, the findings should be treated accordingly. Moreover, since only staff views were explored, future studies could consider also exploring the perspectives of students from the subcontinent.
